# Extraction and Comparative Assessment of the Physicochemical Properties of Pectin From Four Fruit Wastes in Ghana

**DOI:** 10.1155/sci5/9954324

**Published:** 2025-12-17

**Authors:** Prince George Junior Acquah, Mariam El Boakye-Gyasi, Frederick William Akuffo Owusu, Desmond Asamoah Bruce Otu, Raphael Johnson, Marcel Tunkumgnen Bayor

**Affiliations:** ^1^ Department of Pharmaceutics, Faculty of Pharmacy and Pharmaceutical Sciences, Kwame Nkrumah University of Science and Technology, Kumasi, Ghana, knust.edu.gh

**Keywords:** *Fruit waste*, *FT-IR*, *principal component analysis (PCA)*, *rapid-setting pectin*, *valorisation*

## Abstract

Substantial amounts of waste and by‐products are generated annually from the fruit processing industry – an issue with detrimental environmental, socioeconomic and health impacts. Concerted efforts are currently geared towards curtailing this canker, particularly waste valorisation into eco‐friendly, economical and sustainable biopolymers such as pectin. This study focused on the extraction and comparative analysis of the physicochemical properties of pectin from four fruit wastes: *Mangifera indica* L. (Keitt variety), *Carica papaya* (Solo variety), *Ananas comosus* (MD2 variety) and *Citrus limon* (Eureka variety). Pectin was extracted using the conventional citric acid and NaOH processes. Subsequently, the yield, proximate contents and physicochemical properties of the extracted pectins were analysed and compared. The orthogonal data transformation tool, principal component analysis (PCA), was used to highlight relationships between the extracted pectins. Rapid‐setting high methoxyl (DE > 72%) and pharmaceutical‐grade pectins were extracted (yield ranging from 6.72% to 26.60%) irrespective of the extraction method. All the pectins conformed to high‐quality standards (anhydrouronic acid content > 60%, ash content < 2% and moisture content < 5%). FT‐IR analysis revealed that the primary structure of pectin was maintained in all samples. Moreover, all the pectins were sparingly soluble in water (25°C) and exhibited statistically significant (*p* < 0.0001) variations in the swelling and water‐holding properties. PCA demonstrated the clustering of pectins from the same sources despite extraction techniques, highlighting that despite the variations, pectins from the same source exhibit some degree of similarity. The present study presents alternative high‐quality pectins with good physicochemical properties that can be utilised in pharmaceutical dosage forms.

## 1. Introduction

The global fruit processing industry, particularly in sub‐Saharan Africa, has seen a major boost primarily due to increased demands for on‐the‐go fresh foods and lifestyle changes [1]. In 2019, the Food and Agriculture Organisation reported that an excess of 883 million tonnes of fresh fruits was produced [2]. Nonetheless, huge amounts of these fruits (1.6 billion tons) valued at €800 billion and representing a third of the annual global food production are lost as waste [3, 4]. In Ghana, significant proportions of postharvest losses (more than 40%) are recorded in the pineapple, mango and citrus industries [5]. These tonnes of waste yield significant quantities of suspended solids and toxic gases, which adversely impact the environment [6, 7].

Sagar and colleagues reported the rich bioactive components and functional properties of fruit and vegetable wastes [8]. Consequently, research has focused on the valorisation of fruit wastes to provide cost‐effective, eco‐friendly and sustainable resources. A promising industry, particularly for pharmaceutical and drug development research, is the extraction of biopolymers from these organic wastes [9–11].

Pectin is a heteropolysaccharide polymer naturally present as a complex in a plant’s primary cell wall [12]. The source, extraction method and other environmental factors significantly impact its structure (functional groups), physicochemical characteristics (such as degree of esterification [DE] and molecular weight) and functional applications [8–11]. In the food industry, low‐methoxyl pectins (with a DE less than 50%) are commonly used as effective stabilisers in dairy products. Drugs intended for oral administration, such as tablets, gels, hydrogels, beads, aerogels, suspensions and matrix‐controlled dosage forms, are also formulated with pectin [13–20]. Furthermore, pectin has been investigated in colon‐specific medication delivery systems due to the activity of pectinolytic hydrolysing enzymes in the colon [21]. Industrially, Nsom and colleagues reported the adsorbent activity of pectin in the textile industry [22]. Pectin has also been employed as a fluoride absorbent as part of a biomaterial scaffold by Raghav et al. [23].

The conventional acid extraction technique is routinely employed in pectin isolation; nevertheless, the high hydrolysing and depolymerising properties of inorganic acids (HCl, HNO_3_) render the mild organic acids (citric acids) the solvents of choice [24]. Moreover, it is reported that alkaline extraction techniques, despite their effectiveness, yield pectin of low molecular weight and methoxyl content (MeC) due to *β*‐elimination and saponification reactions; as such, they are better suited for oligosaccharide production [25].

*Mangifera indica* L., *Carica papaya*, *Ananas comosus* and *Citrus limon* have been reported to contain considerable amounts of cellulose, hemicelluloses and pectin (both high and low methoxyl), presenting suitable alternative pectin sources [26–28]. However, there is a paucity of literature on a comprehensive comparison of pectin from these sources and the effect of different conventional extraction techniques (acid and alkaline extraction) on the physicochemical parameters. This research seeks to investigate novel sources of quality pectin and its physicochemical properties against this background.

## 2. Methods

### 2.1. Materials

Sodium hydroxide (NaOH) pellets (Merck, Catalogue No. 106498), petroleum ether (Sigma‐Aldrich, Catalogue No. 320447) and distilled water were obtained from the chemical stores of the Department of Pharmaceutics Laboratory, Kwame Nkrumah University of Science and Technology (KNUST), Kumasi, Ghana. Analytical‐grade concentrated hydrochloric acid (HCl) (Sigma‐Aldrich, Catalogue No. 320331), citric acid (Merck, Catalogue No. 100244), ethanol (96%) (Merck, Catalogue No. 100983), chloroform (Sigma‐Aldrich, Catalogue No. 288306), methanol (Merck, Catalogue No. 106009) and sodium chloride (NaCl) (Merck, Catalogue No. 106404) were procured from UK Chemicals, Kumasi, Ghana. All other reagents, unless otherwise specified, were of analytical grade and were also sourced from UK Chemicals, Kumasi, Ghana.

### 2.2. Sample Collection and Identification

Fresh peels of fully ripe *Mangifera indica* L. (Keitt variety), *Carica papaya* (Solo variety), *Ananas comosus* (MD2 variety) and *Citrus limon* (Eureka variety) were randomly acquired from retail fruit stalls at the Ayigya Market (Tech Junction), Kumasi. Approximately 40 kg of peels cut approximately 1 mm from the pulp were collected and authenticated at the Department of Herbal Medicine, KNUST, by Mr. Clifford Asare, the department Herbalist.

Voucher specimen numbers (Table 1) were assigned and kept at the Faculty of Pharmacy and Pharmaceutical Sciences, KNUST Herbarium.

**Table 1:** Voucher specimen numbers of fresh peels.

Specimen	Voucher number
Mango	KNUST/HMI/2023/F007
Pawpaw	KNUST/HMI/2023/F008
Pineapple	KNUST/HMI/2023/F009
Lemon	KNUST/HMI/2023/F010

### 2.3. Sample Preparation and Extraction

The foreign matter associated with the peels was thoroughly removed under running water. The peels were cut into pieces and sun‐dried for 7 days until they were crisp and of constant weight. Subsequently, the dried peels were pulverised into fine powder using a compact blender (ARMG Ardee, India). Each sample was divided into two equal portions and stored in a labelled airtight ziplock bag until use. The method reported by Owusu et al. [29] was adapted for acid and alkaline extraction of pectin. Ground peels (150 g) of mango, pawpaw, pineapple and lemon were carefully weighed into separate 500 mL volumetric flasks.

For acid extraction, 250 mL of acidified water (pH adjusted to 1.5 with citric acid) was added to the volumetric flasks and heated at 84°C for 4 h. The mixture was filtered using a cheesecloth, followed by the addition of 250 mL of 96% ethanol to 100 mL of filtrate (2.5:1) to precipitate pectin. Precipitation was carried out for 30 min, after which the gelatinous pectin precipitates were decanted into a porcelain dish. This process was repeated until no new precipitates formed. The pectin in the porcelain dishes was dried in a hot‐air oven at 30°C until a constant weight was achieved.

The same amount of ground peels (150 g) and extraction conditions (temperature (84°C) and time (4 h)) were maintained for the alkaline extraction processes. The only variation was the use of 250 mL alkaline water (pH adjusted to 9.0 with NaOH) as the extracting solvent.

The samples were stored separately in an air‐tight ziplock bag in a cool and dry place and coded as *MA, ML, PAA, PAL, PIA, PIL, LA and LL* for acid‐extracted mango pectin, alkaline‐extracted mango pectin, acid‐extracted pawpaw pectin, alkaline‐extracted pawpaw pectin, acid‐extracted pineapple pectin, alkaline‐extracted pineapple pectin, acid‐extracted lemon pectin and alkaline‐extracted lemon pectin, respectively. The percentage yield (Y %) was determined on a dry weight basis using Equation (1).

(1) Y%=Weight of pectin extracted gWeight of dried powdered fruit peel 150 g×100.

### 2.4. Proximate Content

The methods described by Bhardwaj et al. [30] were adopted with minor modifications. All determinations were performed in triplicate.

#### 2.4.1. Crude Fat Content

Pectin (1 g) was weighed into an extraction thimble and placed in a Soxhlet apparatus. A dry preweighed solvent flask was attached beneath the apparatus, after which 150 mL of petroleum ether was added. The setup was connected to a condenser and was extracted for 2 h. The thimble and ether were reclaimed after the extraction was completed. Complete removal of the ether was ensured by heating the flask in a boiling bath at 105°C for 30 min. After cooling in a desiccator, the percentage of crude fat was calculated using Equation (2):

(2) % crude fat=Weight of fatWeight of pectin×100,

where weight of fat = final weight of flask − Initial weight of flask

#### 2.4.2. % Crude Fibre Content

Two grams (2 g) of fat‐free and dried pectin were transferred to a digestion flask. Hot H_2_SO_4_ was added to the flask, and the mixture was boiled for 30 min under a condenser. The mixture was immediately filtered through linen and washed thoroughly with boiling water. The residue was transferred back into a digestion flask and mixed with 200 mL of hot NaOH solution. The same process was repeated; however, filtration was performed through a porous crucible and washed with boiling water and 15 mL of 95% ethanol. The residue was dried to a constant weight at 105°C. The dried residue was incinerated at 550°C for 30 min, cooled and weighed again. % Crude fibre was determined using Equation (3).

(3) % crude fibre=Weight of dried residue−weight of ashed residueWeight of pectin×100.

#### 2.4.3. % Total Ash Content

Equation (4) was used to calculate the % ash after 2 g of pectin was incinerated in a muffle furnace at 550°C for 4 h. A dried and previously weighed porcelain dish was used.

(4) % Total Ash=Weight of ashWeight of pectin×100,

where weight of Ash = final weight of dish − Initial weight of empty dish

#### 2.4.4. % Moisture Content (MC)

One gram (1 g) of pectin was weighed into a moist dish and dried to a constant weight at 105°C in a hot‐air oven. Equation (5) was used to determine the MC.

(5) % MC=Initial weight of pectin−Weight of dried pectinInitial weight of pectin×100.

#### 2.4.5. % Crude Protein Content (PRO)

The Kjeldahl method, which involves digestion, distillation and titration, was used to determine the PRO [31].

Equation (6) was used to determine the percentage of crude protein.

(6) % Crude protein=Total Nitrogen×Protein factor.

The Total Nitrogen was determined using Equation (7):

(7) %N=14100×A−B×N×10000.2×,

where N, A and B represent the normality and volume of standard HCl used in the test and blank titrations, respectively. The protein factor is 6.25.

#### 2.4.6. % Nitrogen‐Free Extract (NFE) Carbohydrate Content (% NFE)

The NFE carbohydrate was determined using Equation (8)

(8) %NFE=100−% CP+% CF+% total ash+% CL,

where CP, CF and CL represent the crude protein, crude fibre and crude lipid, respectively.

### 2.5. pH of Pectin

The pH of the 1% sonicated pectin solution was determined using a calibrated Eutech pH metre (pH 510, pH/mV/°C, SN: 2025520, Singapore) [17]. Triplicate measurements were performed.

### 2.6. Swelling Index (SI) of Pectin

The method described by Boakye‐Gyasi et al. [17] was used. To 10 mL of distilled water in a measuring cylinder, 1 g of pectin was added, stopped, mildly agitated and left to rest on the bench overnight. The initial volumes and volumes occupied after 24 h were noted. The volume change was expressed as a percentage of the initial volume. Triplicate measurements were performed.

### 2.7. Water Holding Capacity (WHC)

The contents of the measuring cylinder in Section 2.6 were filtered into a dry 10‐mL measuring cylinder. The volume of water drained into the measuring cylinder was recorded in triplicate. The WHC was calculated as the difference between the initial volume of mucilage and the volume of water drained.

### 2.8. Solubility

The solubility of pectin samples was determined in polar and nonpolar solvents specifically; water (55°C and 25°C), petroleum ether, ethanol, methanol and chloroform. One (1) *g* of pectin was weighed in a 250‐mL beaker already containing 50‐mL solvent, covered with aluminium foil and left to stand overnight on the bench at room temperature. In a preweighed Petri dish, 25 mL of the supernatant was measured, evaporated and dried to constant weight over a water bath. The residual weight determined was represented as a percentage of the solubility of the pectin in the individual solvents [17]. Triplicate determinations were made for all pectin samples.

### 2.9. Characterisation of Pectin

#### 2.9.1. Fourier Transform Infrared (FTIR) Spectra Analysis

Spectra of pectin samples were generated using the FTIR spectrophotometer (PerkinElmer, UATR Spectrum 2, 94133, UK). The wavelength range scanned was 400–4000 cm^−1^ at 4 cm^−1^ resolution. The multiple spectra generated after scanning each pectin sample 24 times were merged using Origin Pro 2024 (OrignLab, Massachusetts, USA).

#### 2.9.2. Equivalent Weight (EQ)

Equation (9) was used to determine EQ after the titrimetric method described by Owusu et al. [29] was carried out. Briefly, 0.5 g of pectin was transferred into a 250‐mL conical flask and moistened with ethanol (5 mL). Additionally, 1 g of NaCl, 100 mL of distilled water and 6 drops of phenol red indicator were added and thoroughly mixed to ensure uniformity. Titration to a purple endpoint was carried out by titrating the mixture against 0.1N NaOH.

(9) EQ=Weight of pectin×100Volume of NaOH×Nomality of NaOH.

#### 2.9.3. MeC

To the neutralised solution in Section 2.9.2, 25 mL of 0.25 N NaOH was mixed thoroughly at 25°C for 30 min. The solution was titrated against 0.1 N NaOH after drops (5) of phenol red and 25 mL of 0.25 N HCl were added. The endpoint was a faint pink colouration [29]. MeC was calculated from Equation (10).

(10) MeC=Volume of NaOH×Nomality of NaOH×31100× Weight of pectin.

The molecular weight of the methoxyl group is 31.

#### 2.9.4. Anhydrouronic Acid Content (AUA)

The values obtained from Sections 2.9.2 and 2.9.3 were inputted into Equation (11) to determine the AUA [29].

(11) %AUA=176100×weight of pectin/µeq equivalent weight+MeC.

The molecular weight of AUA is 176.

#### 2.9.5. DE

The values acquired from Sections 2.9.2–2.9.4 were inputted into Equation (12) to determine the DE.

(12) DE=MeC×176100×AUA×31.

##### 2.9.5.1. DE Using FTIR Spectra

The absorbance densities (A) accounted for by the free carboxyl groups (1744.09 cm^−1^) and esterified carboxylic (1629.85 cm^−1^) were inserted into Equation (13) to obtain the DE [32, 33].

(13) DE=124.72.2013× A1744.09A1744.09+A1629.85+.

### 2.10. Data Analysis

Experimental data was inputted in Microsoft Excel and screened for consistency and accuracy before being analysed with GraphPad Prism version 8.0.1 (GraphPad Software, San Diego California, USA). Bar charts, frequency tables, Tukey’s and scatter plots were used to represent data using the mean ± standard deviation (SD). The principal component analysis (PCA) of the standardised data was carried out using Origin Pro 2024 (OrignLab, Massachusetts, USA). The *p-value* was set at an *α* threshold of 5%. The Shapiro–Wilk and Brown–Forsythe tests for normality and homoscedasticity, respectively, were evaluated before assumptions of Gaussian distribution and equal variances were made.

## 3. Results and Discussion

### 3.1. Percentage Yield

The yield of pectin has been reported to be affected by a myriad of factors including but not limited to the method and time of extraction, particle size, source, solid–liquid ratio, pH and type of solvent [25, 28, 34]. The conventional heating technique (at 75°C–100°C for 1–3 h) utilised in the commercial production of pectin has been reported with variable success [28, 35]. However, organic acids (e.g. citric acid) compared to mineral acids (e.g. HCl and HNO_3_) are less depolymerising and hydrolysing when solubilising pectin from protopectin and other cellulose microfibrils at high temperatures [25, 28, 35]. Previous studies utilising citric have reported yields ranging from 5.3% to 6.2% on dry‐weight bases, which were higher than when HCl was used [28]. The high dissociation constant of HCl results in the generation of relatively higher amounts of hydroxonium ions (H_3_O^+^), leading to pectin depolymerisation and lower yields [28, 35]. Compared to the acids, there is an increased tendency for pectin degradation via *β*‐elimination (cleavage of glycosidic bonds) and saponification reactions when NaOH is used [28, 35]. However, the work by Wandee and colleagues highlighted that this may not impact the yield because relatively higher yields were obtained for different concentrations of NaOH (13.9%–24.2%) when compared to HCl (6.5%–20.5%) [25].

**Figure 1:**
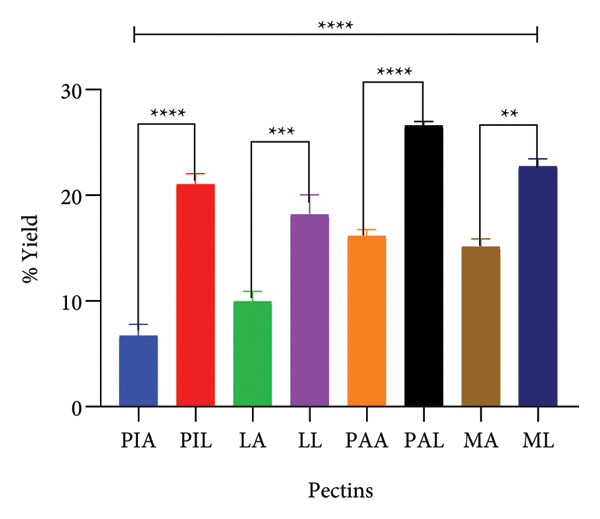
Percentage yield of pectin samples. Graphs were generated using mean ± SD (*n* = 3); ^∗∗^ *p* < 0.01; ^∗∗∗^ *p* < 0.001: ^∗∗∗∗^ *p* < 0.0001 significant difference between acid (A) and alkaline (L) extraction techniques and between different pectin sources (ordinary one‐way ANOVA followed by Tukey’s multiple comparisons test). MA, ML, PAA, PAL, PIA, PIL, LA and LL are acid‐extracted mango pectin, alkaline‐extracted mango pectin, acid‐extracted pawpaw pectin, alkaline‐extracted pawpaw pectin, acid‐extracted pineapple pectin, alkaline‐extracted pineapple pectin, acid‐extracted lemon pectin and alkaline‐extracted lemon pectin, respectively.

The results (in Figure 1) obtained (6.72 ± 1.85 (PIA) to 26.60 ± 0.66 (PAL)) were comparable to other studies. Specifically, Karim et al. [36] reported a yield of 3.88%–13.06% for pineapple, which was confirmed by Shivamathi and colleagues who obtained a maximum yield of 16.24% [36, 37]. Similarly, a maximum yield of 23.69% was obtained for mango peels by Lai and colleagues, while another study reported a yield between 18.8% and 32.1% [38, 39]. For pawpaw and lemon, a maximum yield of 23.74% and 25.31%, respectively, have been reported [40, 41]. A significantly higher yield was obtained from the alkaline extraction process compared to the acidic process which was comparable to the results by Wandee et al. [25]. This observation may be explained at least in part by the pH of extraction as indicated by Chan et al. [35]. At lower pH (below 4–5), pectin molecules are hydrolysed to greater extents in acidic media compared to alkaline media. Although the argument for the depolymerisation of pectin via *β*‐elimination may be made, this process is highly temperature‐dependent (maintained at 84°C); thus, the hydrolysis of pectin by the acid may offer the most plausible explanation for the difference [35].

### 3.2. Proximate Content

The proximate content is a major determinant of the purity of pectin and is often employed in the quality control process. All the pectins extracted passed the proximate content albeit with some significant differences (*p* < 0.05) between them (Table 2). LL, PIL, PAA, MA, PAL and PIA recorded higher percentages of fats, crude fibre, ash contents, moisture, protein and NFE carbohydrates, respectively.

**Table 2:** Proximate content of pectin from different fruit sources and extraction techniques.

Pectin	Proximate content (%)					
	Fat	Crude fibre	Total ash	Moisture	Protein	NFE carbohydrate
PIA	4.00 ± 0.23^ns^	0.51 ± 0.05^c^	0.65 ± 0.03^ns^	2.38 ± 0.09^ns^	7.10 ± 0.10^ns^	85.36 ± 0.71^g^
PIL	4.20 ± 0.10^ns^	0.78 ± 0.07 ^d^	0.77 ± 0.02^ns^	2.45 ± 0.05^ns^	7.48 ± 0.10^ns^	84.32 ± 0.18^f^
LA	3.80 ± 0.08^a^	0.46 ± 0.05^ns^	1.09 ± 0.02^ns^	2.50 ± 0.07^g^	10.22 ± 0.11^a^	81.93 ± 0.35^a^
LL	5.00 ± 0.23^b^	0.49 ± 0.04^ns^	1.05 ± 0.04^ns^	2.00 ± 0.14h	11.68 ± 0.17^b^	79.78 ± 0.27^b^
PAA	3.00 ± 0.23^a^	0.51 ± 0.04e	1.28 ± 0.05^ns^	2.58 ± 0.11^ns^	10.95 ± 0.12^a^	81.68 ± 0.19^a^
PAL	4.00 ± 0.19^b^	0.69 ± 0.07f	1.21 ± 0.11^ns^	2.44 ± 0.34^ns^	12.56 ± 0.29^b^	79.10 ± 0.13^b^
MA	2.00 ± 0.10^ns^	0.24 ± 0.02^ns^	0.91 ± 0.02^a^	3.12 ± 0.03^c^	5.71 ± 0.10^ns^	88.02 ± 0.14^ns^
ML	2.10 ± 0.05^ns^	0.27 ± 0.04^ns^	1.18 ± 0.03^b^	2.35 ± 0.07 ^d^	5.31 ± 0.08^ns^	88.79 ± 0.21^ns^

The mean ± SD of triplicate (*n* = 3) determinations are presented; different alphabets after the same pectin source (PI, L, PA and M) represent a significant difference between the acid (A) and alkaline (L) extraction techniques (*p* < 0.0001 (a and b), *p* < 0.001 (c and d), *p* < 0.01 (e and f) and *p* < 0.05 (g and h), *p* > 0.05 nonsignificant (ns), using ordinary one‐way ANOVA followed by Tukey’s multiple comparisons test. MA, ML, PAA, PAL, PIA, PIL, LA and LL are acid‐extracted mango pectin, alkaline‐extracted mango pectin, acid‐extracted pawpaw pectin, alkaline‐extracted pawpaw pectin, acid‐extracted pineapple pectin, alkaline‐extracted pineapple pectin, acid‐extracted lemon pectin and alkaline‐extracted lemon pectin, respectively.

#### 3.2.1. Moisture Content

The MC has a multifactorial impact on pectin quality as a functional product. From a microbial quality focus, lower MC (< 15% according to the BP and < 12% according to the IPPA) discourages the growth of mould and pathogenic microbes which ensures product quality during its shelf‐life [42, 43]. Aziz and colleagues opined that values < 5% indicate an efficient drying process and assure microbial safety [44]. However, storage in an airtight container is still required due to the hygroscopic nature of pectin. Additionally, high MC can impact the pectin’s overall quantity, reducing its market value after drying. Moisture content also impacts the flow properties, stickiness and bulk density of powders [45–47]. A positive correlation has been observed in tabletting between MC and bulk density due to the increased interparticulate interactions among powder particles [44]. Indeed, high moisture strengthens the van der Waals forces and decreases electrostatic forces leading to high cohesiveness and stronger liquid bridges [44, 48]. Crouter and Briens [48] interestingly posited that despite this prevailing dogma, controlled amounts of moisture can increase flow properties by acting as a lubricating fluid. Finally, MC impacts granule size during granulation and also significantly impacts the tensile strength of the final tablet product [48].

#### 3.2.2. Crude Fat

The crude fat represents the amount of fat‐soluble materials (phospholipids, steroids, triglycerides, etc.) in the sample [49]. Together with proteins and carbohydrates, they contribute to the total energy content [50]. Additionally, from a pharmaceutical viewpoint, the lipid content impacts the flowability and overall quality of the pectin to be employed as a pharmaceutical excipient. High‐fat content decreases the wettability of the pectin, decreases flowability and increases the risk of pectin oxidation [44].

#### 3.2.3. Protein and Crude Fibre Content

The protein content has also been reported to impact the surface morphology of powder, flow properties and the dissolution profile of pharmaceutical dosage forms. High protein contents impact gelatinisation, water retention and interaction with other macromolecules [44]. Moreover, the amphiphilic nature of proteins has been reported to significantly increase the emulsifying properties of pectin by adsorbing at the oil/water interface [51, 52]. However, as indicated by Gawkowska and colleagues, a trade‐off is required as excess amounts of proteins achieve the opposite (variations in pH and decreased stability of emulsions) [53]. The crude fibre contents have also been implicated in flow properties with Saifullah and colleagues reporting that a powder with low fat and high fibre contents tends to exhibit superior flow properties [44, 54].

#### 3.2.4. Ash Content and NFE Carbohydrate

The purity of pectin with regards to the absence of insoluble salts, complexes as well as inorganic residues is determined by the ash content. Lower values (< 10%) are recommended by the International Pectin Producers Association (IPPA) as they impact the quality of pectin [43, 50]. Additionally, the presence of inorganic salts can impact the swelling and gelling properties of the pectin further affecting other downstream functional applications [43, 55, 56]. The very low ash contents and thus purity were supported by the NFE carbohydrate values which represent the total polysaccharides present [50]. A high value indicates a high‐quality pectin devoid of unacceptable contamination. Moreover, it can be inferred that the extraction process was appropriate and optimised the extraction of high‐quality pectin.

### 3.3. pH

**Figure 2:**
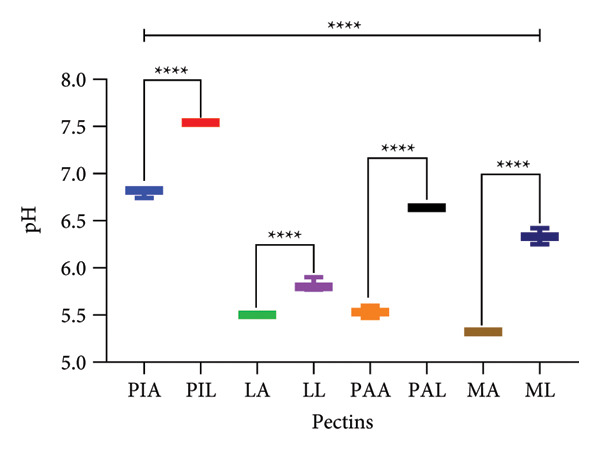
pH of 1%w/v pectin solutions. Graphs were generated using mean ± SD (*n* = 3); ^∗∗∗∗^ *p* < 0.0001 significant difference between acid (A) and alkaline (L) extraction techniques and between different pectin sources (PI, L, PA, M) (ordinary one‐way ANOVA followed by Tukey’s multiple comparisons test). MA, ML, PAA, PAL, PIA, PIL, LA and LL are acid‐extracted mango pectin, alkaline‐extracted mango pectin, acid‐extracted pawpaw pectin, alkaline‐extracted pawpaw pectin, acid‐extracted pineapple pectin, alkaline‐extracted pineapple pectin, acid‐extracted lemon pectin and alkaline‐extracted lemon pectin, respectively.

The pH of the samples significantly (*F*_7,16_ *= 746.5,* *p* < 0.0001) ranged from slightly acidic (5.32 ± 0.09 for MA) to neutral (7.54 ± 0.01 for PIL). Consistently, the alkaline extraction techniques yielded significantly (*p* < 0.0001) higher pH when compared to the acid extraction technique (Figure 2). Pectin polysaccharides are anionic and therefore liable to pH variations in pectin dispersions [35]. Lower pH values protonate carboxylic acid groups and promote conformational changes to a more compact three‐fold helical pectin. Subsequently, electrostatic repulsions between and along the pectin network decrease, while associations with secondary alcohols increase leading to the formation of inter‐ and intramolecular hydrogen bonds. Indeed, these interactions culminate in the dimerisation of the pectin networks, immobilisation of water and finally, gelation [28, 57, 58]. In high‐methoxyl pectin (DE > 50%), gelation occurs at lower pH ranges (< 3.5) in the presence of co‐solutes like sucrose while low‐methoxyl pectins (DE < °50%) gel over a wider range of pH (2–6) [28, 57, 58]. Several studies have reported the impact of pH on pectin gelation [59–61]. The results obtained conform to those reported by Owusu et al. [62] (6.39–6.92) who discussed that the slightly acidic to neutral pH prevents untoward irritation of the gastrointestinal mucosa and may not affect the release of any active pharmaceutical ingredient incorporated in the pectin networks.

### 3.4. SI and WHC of Pectin

Figure 3 shows the SIs (a) and WHCs (b) of pectin samples. Significant differences (*p* < 0.0001) were observed for the SIs of the various pectin samples when the different sources and extraction methods were compared. PAA and PAL swelled to about 90% of their original size while LA recorded the least SI (25% ± 0.02). PIA and PIL also demonstrated the highest WHCs (7 mL).

Figure 3(a) Swelling index and (b) water‐holding capacity of pectin samples. Graphs were generated using mean ± SD (*n* = 3); ^∗^ *p* = 0.0447, ^∗∗∗∗^ *p* < 0.0001 significant difference between acid (A) and alkaline (L) extraction techniques and between different pectin sources (PI, L, PA, M) (ordinary one‐way ANOVA followed by Tukey’s multiple comparisons test). MA, ML, PAA, PAL, PIA, PIL, LA and LL are acid‐extracted mango pectin, alkaline‐extracted mango pectin, acid‐extracted pawpaw pectin, alkaline‐extracted pawpaw pectin, acid‐extracted pineapple pectin, alkaline‐extracted pineapple pectin, acid‐extracted lemon pectin and alkaline‐extracted lemon pectin, respectively.

**Figure:**
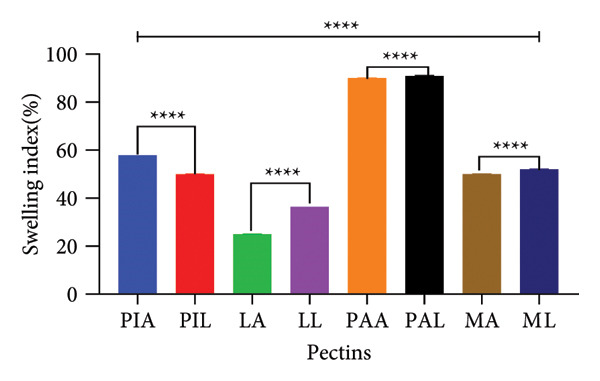
(a)

**Figure:**
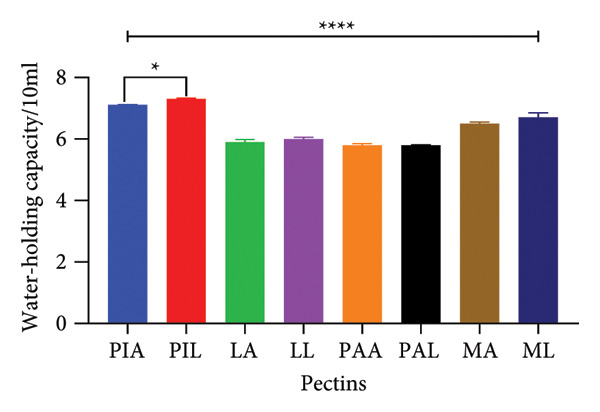
(b)

Natural biopolymers imbibe and dissolve or swell in water due to the hydrophilic functional groups present such as the galacturonic acid groups in pectin [63]. The quantum of water imbibed when no external stressors are applied indicates the WHC and swelling properties and they play a critical role in the functional applications of the biopolymer [64–66]. Both properties have been reported to be affected by variations in pH, ionic strength and the structure of pectin [37, 65]. Boakye‐Gyasi and colleagues argued that these properties are implicated in the hydration and gelling properties of pectin which also inform their use in controlled‐release formulations [17]. Additionally, the gelling ability afforded by the swelling and water‐holding properties of pectin makes it ideal to be used as a viscosity modifier in oral dosage forms. The values obtained were less than those reported by Adi‐Dako and colleagues for cocoa pod husk pectin (360%) and Boakye‐Gyasi et al. [17] (300%–500%) for okra pectin; however, compared to other works, the results were higher, indicating the variability associated with different pectin sources and extraction processes [17, 46, 67].

### 3.5. Solubility

Generally, the pectin samples demonstrated practical insolubility in pet ether and chloroform (Tables 3 and 4). In methanol, ethanol, water (55°C), and water (25°C), the samples showed varying amounts of solubility but none was very soluble in either solvent. The method of extraction also impacted significantly (*p* < 0.05) the extent of solubility.

**Table 3:** Solubility of pectin in common solvents.

Pectin	Solubility (%)					
	Pet ether	Water (@ 55°C)	Water (@ 25°C)	Ethanol (96%)	Methanol	Chloroform
PIA	—	172.12 ± 1.7^a^	90.89 ± 2.6^ns^	1540 ± 0.5^a^	92.59 ± 1.0^e^	—
PIL	—	192.59 ± 5.7^b^	89.28 ± 1.7^ns^	1250 ± 0.2^b^	86.58 ± 5.7^f^	—
LA	—	38.00 ± 1.5^a^	166.67 ± 2.6^a^	625 ± 0.5^a^	251.08 ± 0.8^a^	—
LL	—	87.68 ± 0.2^b^	1059.8 ± 2.5^b^	487 ± 2.9^b^	208.33 ± 0.3^b^	—
PAA	—	89.03 ± 0.5^a^	35.78 ± 9.5^ns^	1250 ± 0.3^a^	178.57 ± 3.8^a^	18657.3 ± 0.1^a^
PAL	—	52.7 ± 2.8^b^	34.77 ± 8.9^ns^	1468 ± 2.6^b^	267.89 ± 0.6^b^	19639.1 ± 0.2^b^
MA	—	73.12 ± 1.3^ns^	60.24 ± 1.4^g^	119.05 ± 0.4^a^	85.89 ± 0.3^a^	—
ML	—	74.71 ± 8.5^ns^	65.79 ± 2.7^f^	178.58 ± 0^b^	98.56 ± 0.4^b^	42090.3 ± 0.1

*Note:* — = no recovered amounts after drying.

The mean ± SD of triplicate (*n* = 3) determinations is presented; different alphabets after the same pectin source (PI, L, PA and M) represent significant differences between the acid (A) and alkaline (L) extraction techniques (*p* < 0.0001 (a and b), *p* < 0.01 (e and f) and *p* < 0.05 (g and h), *p* > 0.05 nonsignificant (ns), using ordinary one‐way ANOVA followed by Tukey’s multiple comparisons test. MA, ML, PAA, PAL, PIA, PIL, LA and LL are acid‐extracted mango pectin, alkaline‐extracted mango pectin, acid‐extracted pawpaw pectin, alkaline‐extracted pawpaw pectin, acid‐extracted pineapple pectin, alkaline‐extracted pineapple pectin, acid‐extracted lemon pectin and alkaline‐extracted lemon pectin, respectively.

**Table 4:** The solubility of pectin in common solvents using the BP and USP criteria.

Pectin	Solvents					
	Pet ether	Water (@ 55°C)	Water (@ 25°C)	Ethanol (96%)	Methanol	Chloroform
PIA	Practically insoluble	Slightly soluble	Sparingly soluble	Very slightly soluble	Sparingly soluble	Practically insoluble
PIL	Practically insoluble	Slightly soluble	Sparingly soluble	Very slightly soluble	Sparingly soluble	Practically insoluble
LA	Practically insoluble	Sparingly soluble	Slightly soluble	Slightly soluble	Slightly soluble	Practically insoluble
LL	Practically insoluble	Sparingly soluble	Slightly soluble	Slightly soluble	Slightly soluble	Practically insoluble
PAA	Practically insoluble	Sparingly soluble	Sparingly soluble	Very slightly soluble	Slightly soluble	Practically insoluble
PAL	Practically insoluble	Sparingly soluble	Sparingly soluble	Very slightly soluble	Slightly soluble	Practically insoluble
MA	Practically insoluble	Sparingly soluble	Sparingly soluble	Slightly soluble	Sparingly soluble	Practically insoluble
ML	Practically insoluble	Sparingly soluble	Sparingly soluble	Slightly soluble	Sparingly soluble	Practically insoluble

MA, ML, PAA, PAL, PIA, PIL, LA and LL are acid‐extracted mango pectin, alkaline‐extracted mango pectin, acid‐extracted pawpaw pectin, alkaline‐extracted pawpaw pectin, acid‐extracted pineapple pectin, alkaline‐extracted pineapple pectin, acid‐extracted lemon pectin and alkaline‐extracted lemon pectin, respectively.

The evaluation of the behaviour of polymers in different solvents is best determined by solubility [45]. Solubility only ensues after the polymers have been adequately dispersed and wetted, and it is affected by changes in temperature, pH, particle size and co‐solvents [45, 64]. The percentage solubilities of pectin in the hydrophilic polar solvents were higher than those in the hydrophobic nonpolar solvents highlighting the polar nature of pectin (Tables 3 and 4). In polar solvents, the carboxylic acid groups of pectin are dissociated which enhances the interaction with solvent molecules. Additionally, when the temperature of the water was raised from 25°C to 55°C, an increase in solubility was achieved. This could be accounted for by the increased mobility (and interactions) afforded by the acquisition of kinetic energy [68]. The solubility pattern of the pectin samples was similar to those previously reported [13, 62]. Moreover, because of the sparing solubility of the pectin extracts in water coupled with the appreciable water‐holding and swelling properties, the pectin samples can act as good viscosity modifiers [69].

### 3.6. Characterisation of Pectin

#### 3.6.1. EQ

The EQ of pectin represents the total content of free or unesterified galacturonic acids which can cross‐link with polyol (‐OH) functional groups [11, 70, 71]. It is an essential characterisation parameter which correlates highly with the gelling properties and other functional applications of pectin. Additionally, it is an indicator of pectin purity and the efficiency of the extraction process [11, 70, 71]. Low EQ values imply partial degradation reactions of the pectin after its solubilisation [40, 70, 72]. Several papers postulate that lower extraction pH aids in pectin polymerisation leading to higher EQs as observed for the mango and pineapple pectins (Table 5) [71, 73]. Nevertheless, other schools of thought indicate that lower pH extraction procedures may lead to hydrolysis and partial degradation of pectin chains into weaker networks thus decreasing the EQ as observed for the lemon and pawpaw pectins [35, 74, 75]. Generally, high EQs were obtained for the pectin samples when compared to other pectins (641%–2500%), commercial citrus (1190%) and apple (515%) pectins, and the recommended minimum amounts by the IPPA (400%) indicating superior gelling properties of the pectin extracts [27, 39, 70]. The significant difference also supports the assertions that different raw materials and extraction materials influence the EQ [11].

**Table 5:** Characterisation of pectin from different fruit sources and extraction techniques.

Pectin	Parameter (%)			
	Equivalent weight	Methoxyl content	Anhydrouronic acid content	Degree of esterification
PIA	3229.0 ± 147.30^ns^	10.88 ± 0.04^a^	67.23 ± 0.50^a^	91.89 ± 0.31^ns^
PIL	3452.0 ± 168.40^ns^	11.84 ± 0.09^b^	72.34 ± 0.25^b^	92.94 ± 0.36^ns^
LA	4583.0 ± 589.30^a^	12.06 ± 0.04^a^	72.34 ± 0.75^a^	94.65 ± 0.63^g^
LL	6696.0 ± 631.30^b^	11.25 ± 0.13^b^	66.53 ± 0.50^b^	96.03 ± 0.40^h^
PAA	1962.0 ± 54.39^c^	11.25 ± 0.13^ns^	72.86 ± 0.10^a^	87.68 ± 0.17^a^
PAL	3709.0 ± 194.30^d^	10.88 ± 0.04^ns^	66.53 ± 0.01^b^	92.86 ± 0.37^b^
MA	1887.0 ± 50.36^ns^	10.08 ± 0.22^c^	66.53 ± 0.10^a^	85.97 ± 0.58^a^
ML	1450.0 ± 29.71^ns^	10.73 ± 0.26^d^	73.04 ± 1.74^b^	83.37 ± 0.06^b^

The mean ± SD of triplicate (*n* = 3) determinations are presented; different alphabets after the same pectin source (PI, L, PA and M) represent a significant difference between the acid (A) and alkaline (L) extraction techniques (*p* < 0.0001 (a and b), *p* < 0.001 (c and d) *p* < 0.05 (g and h) and *p* > 0.05 nonsignificant (ns), using ordinary one‐way ANOVA followed by Tukey’s multiple comparisons tests. MA, ML, PAA, PAL, PIA, PIL, LA and LL are acid‐extracted mango pectin, alkaline‐extracted mango pectin, acid‐extracted pawpaw pectin, alkaline‐extracted pawpaw pectin, acid‐extracted pineapple pectin, alkaline‐extracted pineapple pectin, acid‐extracted lemon pectin and alkaline‐extracted lemon pectin, respectively.

#### 3.6.2. MeC and DE

The sugar molecules (galactose, rhamnose, etc.) in pectin possess free OH groups which are capable of methylation [37]. Based on the MeC, pectin can be categorised into two: high‐methoxyl pectin (> 7%) and low‐methoxyl pectin (< 7%) which have different mechanisms of gelation. Similarly, the DE, which also indicates the extent of methyl esterification of the carboxylic acid groups, can be categorised into two: high‐methoxyl pectin (> 50%) and low‐methoxyl pectin (< 50%) [28, 35]. The extent of the methylation impacts a myriad of the functional applications of pectin such as gelation. However, it is recommended that an assessment of the DE should be done in concert with the MeC as the former investigates only the ratio of methanol‐esterified galacturonic acids to the total galacturonic acids present, while the latter accounts for the total amounts of the methoxyl groups. Therefore, lower levels of galacturonic acids may affect the drawing an appropriate inference [72].

Altogether, they impact the gelling strength, setting time and interaction with metal ions in solvents. The MeC also impacts the dispersibility of pectin in aqueous media, the structure of gels as well as the spreadability of gels. The mechanism of gelling is also affected by the MeC and the DE. For instance, low‐methoxyl pectin in the presence of divalent ions (e.g. Ca^2+^) readily cross‐links to form stable gels that are thermo‐irreversible via a model described as egg‐box or junction zones. Notwithstanding the tendency of high‐methoxyl pectins to also form gels via egg‐box structures, their short and compact structures hinder this mechanism. Instead, they gel via nonionic interactions such as hydrogen bonding and hydrophobic interactions with co‐solutes (notably sucrose) at low pH [28, 39, 40].

**Figure 4:**
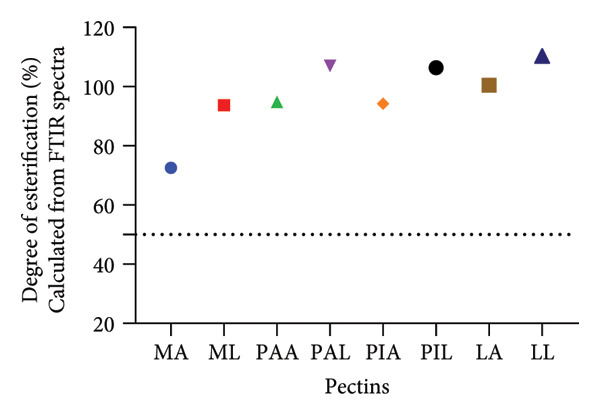
Degree of esterification of pectin samples calculated from the FTIR spectra.

All the pectin samples can be classified as high‐methoxyl pectin (Table 5). Furthermore, based on the gel formation rate classification, they may be described as rapid set pectins as their DE was > 72%. The FTIR method of determining the DE (Figure 4) corroborated this finding as well as other values obtained for commercial pectin sources [39, 40, 75]. Finally, the high MeCs imply that pectin can form stronger gels with strong adhesive and cohesive forces which are ideal characteristics for viscosity enhancers in oral pharmaceutical dosage forms [71].

#### 3.6.3. Anhydrouronic Acid (AUA)

The AUA characterises the amounts of galacturonic acids present in the pectin, and it is essential in determining the pectin purity [39, 40, 76]. For food and pharmaceutical applications, a minimum threshold of 65% is recommended to assure quality. Additionally, higher values correlate with good gelling properties of pectin [27, 72]. The results in Table 5 indicate that all the extracted pectins were not contaminated with high levels of proteins, sugars and other cell wall constituents.

#### 3.6.4. Fourier Transform Infrared (FTIR)

**Figure 5:**
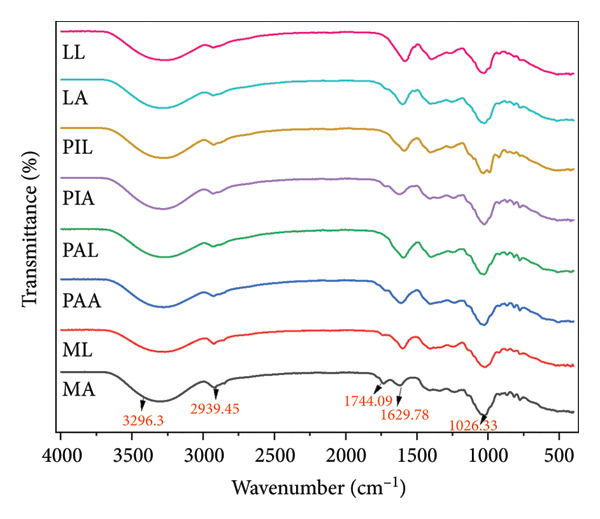
Superimposed FTIR spectra of pectin samples.

**Figure 6:**
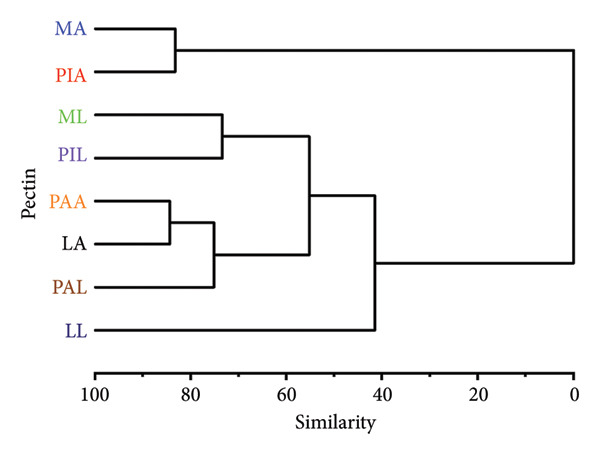
Dendrogram of the percentage transmittance values of pectin samples using agglomerative hierarchical complete linkage cluster analysis. MA, ML, PAA, PAL, PIA, PIL, LA and LL are acid‐extracted mango pectin, alkaline‐extracted mango pectin, acid‐extracted pawpaw pectin, alkaline‐extracted pawpaw pectin, acid‐extracted pineapple pectin, alkaline‐extracted pineapple pectin, acid‐extracted lemon pectin and alkaline‐extracted lemon pectin, respectively.

The FTIR spectra (Figure 5) and the peaks assigned (Table 6) show high levels of similarity between each other, pectin from other sources (Mangosteen and watermelon) and commercial citrus pectin [11, 74, 77]. This was further supported by the dendrogram (Figure 6) highlighting just a single cluster with high similarity (about 80%) [78]. The lack of variations in the FTIR spectra implies that the extraction methods did not denature the primary structure of pectin [25].

**Table 6:** Assignment of peaks and functional observed in the FTIR spectra of pectin samples.

Source of pectin	C‐O stretching of primary alcohol	Methyl‐esterified carboxyl groups	Asymmetric stretching modes of the carboxylate group	Aliphatic C‐H stretching vibrations including CH, CH2 and CH3	O‐H stretching vibrations due to intra‐ and intermolecular hydrogen bonding
	Relevant peaks				
MA	1026.13	1618.48	1730.70	2915.50	3300.84
ML	1022.94	1627.82	1742.05	2924.79	3267.65
PAA	1027.33	1610.82	1740.01	2927.37	3274.44
PAL	1028.79	1611.50	1742.05	2928.24	3276.92
PIA	1026.13	1629.86	1742.05	2928.96	3276.66
PIL	1031.48	1611.50	1742.05	2926.25	3266.23
LA	1026.23	1627.82	1740.01	2929.67	3278.66
LL	1028.08	1629.86	1744.09	2928.66	3265.80

MA, ML, PAA, PAL, PIA, PIL, LA and LL are acid‐extracted mango pectin, alkaline‐extracted mango pectin, acid‐extracted pawpaw pectin, alkaline‐extracted pawpaw pectin, acid‐extracted pineapple pectin, alkaline‐extracted pineapple pectin, acid‐extracted lemon pectin and alkaline‐extracted lemon pectin, respectively.

### 3.7. PCA

The dimensionality reduction algorithm, PCA was used to reduce the dimensions into two components without losing most of the variations in the data set. This orthogonal data transformation ensured easy interpretation of the multidimensional data [70, 79, 80]. The Scree plot (Figure 7(a)) highlights four principal components: PC1, PC2, PC3 and PC4. PC1 contributed to 38.55% of the variation in the physicochemical properties while PC2 contributed to 24.72%, resulting in a cumulative percentage of 63.27%. PC3 and PC4 accounted for 14.86% and 11.85% of the variations, respectively. Generally, the pH, EQ, MeC, crude fibre (FIB), DE, yield (Y), and crude fat (FAT) correlated positively with PC1, while the MC, AUA, MeC, SI, WHC and NFE carbohydrate content (NFEC) negatively correlated with PC1. Furthermore, PC2 accounting for a smaller variation correlated positively with the WHC and NFEC and negatively with MC, PRO and AUA. Clustering of MA and ML occurred in the lower left quadrant, while PIA and PIL clustered in the upper quadrants. LL and LA also clustered on the PC1 axis, while PAA and PAL clustered in the bottom right.

Figure 7Scree plot (a) and PCA biplot (b) of the physicochemical properties of pectin extracts. MA, ML, PAA, PAL, PIA, PIL, LA and LL are acid‐extracted mango pectin, alkaline‐extracted mango pectin, acid‐extracted pawpaw pectin, alkaline‐extracted pawpaw pectin, acid‐extracted pineapple pectin, alkaline‐extracted pineapple pectin, acid‐extracted lemon pectin and alkaline‐extracted lemon pectin, respectively.

**Figure:**
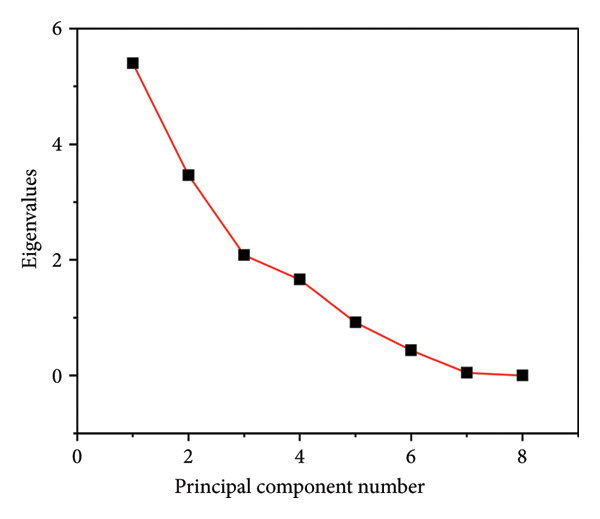
(a)

**Figure:**
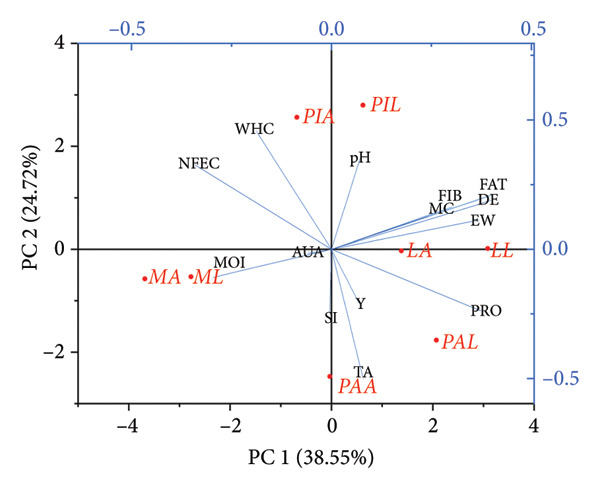
(b)

The biplot (Figure 7(b)) confirmed the earlier discussions that the physicochemical properties of pectin were significantly affected by parameters such as pH, MeC, EQ, SI and proximate contents (strong correlations with PC1 and PC2). The clustering of the acid and alkaline extracted pectins indicated that despite the variations in the extraction method, most of the properties of the pectin are maintained if they are from the same source albeit with significant differences.

## 4. Conclusion

Pectin from four different fruit wastes has been successfully extracted and characterised. The yields from the alkaline extraction technique were high for all fruits compared to the acid extraction process. However, quality rapid‐setting high‐methoxyl pharmaceutical‐grade pectin was extracted by both methods. FT‐IR and agglomerative hierarchical analysis revealed that the primary structure of pectin was maintained in all samples which exhibited good swelling and water‐holding properties despite their sparing solubility in water. The PCA demonstrated the variations in the extracted pectin; nevertheless, clustering of the pectin from the same source pointed to high similarity albeit with significant differences.

## Conflicts of Interest

The authors declare no conflicts of interest.

## Funding

This research did not receive any specific grant from funding agencies in the public, commercial, or not‐for‐profit sectors.

## Data Availability

The information used to support the study’s conclusions is provided in the publication and can also be obtained upon request from the corresponding author.
